# Patient-reported outcomes after breast reconstructive surgery: A prospective cross-sectional study

**DOI:** 10.1016/j.amsu.2019.02.002

**Published:** 2019-02-16

**Authors:** Salem Mohammad Alshammari, Mohammed Yousef Aldossary, Khaled Almutairi, Abdulaziz Almulhim, Gousay Alkhazmari, Mohammed Alyaqout, Hussain Abrar

**Affiliations:** Department of General Surgery, Plastic and Reconstructive Surgery Unit, King Fahad Specialist Hospital, Dammam, Saudi Arabia

**Keywords:** Breast reconstruction, Breast implant, Breast cancer, Patient-reported outcome, Satisfaction, PRO, patient-reported outcome, OPD, out-patient clinic, ABR, autologous breast reconstruction, IBR, implant-based breast reconstruction, IRB, institute of review board, BMI, body mass index

## Abstract

**Background:**

With advancements in and the evolution of the medical field, several methods and surgical techniques have been developed for breast reconstruction after mastectomy. Generally, we can categorize these strategies into two broad groups: autologous reconstruction and implant-based reconstruction. This study aimed to analyze the satisfaction rate between these groups, considering age, timing of breast reconstruction, body mass index (BMI), major complications, and the need for radiotherapy or chemotherapy.

**Materials and methods:**

All the patients who underwent a mastectomy and subsequent breast reconstruction surgery at our institution between August 1, 2013, and August 31, 2017, were invited to complete a BREAST-Q questionnaire. To compare the quality of life and complication rate between the autologous and implant-based reconstruction groups, data were collected from specific patients. All participants completed the Arabic version of the postoperative reconstruction module.

**Results:**

Among 61 patients, 43 (70.5%) completed the two domains of the BREAST-Q questionnaire, about the satisfaction with the implanted breast and satisfaction with the surgical outcome. These patients were divided into two groups: autologous (n = 21) and implant-based (n = 22) groups. The mean score of satisfaction with the implanted breast was 43.5 for the autologous group and 39.6 for the implant-based group. For the surgical outcome, the scores for the autologous and implant-based groups were 45.4 and 56.0, respectively. However, there was neither a statistical significance in the satisfaction with the implanted breast nor the surgical outcome between the two groups.

**Conclusion:**

Although there are many different surgical techniques to reconstruct a breast after mastectomy, there is still no specific surgical method that is perfect or well-suited for all patients undergoing breast reconstruction surgery. In our study, we found that there was no significant difference in satisfaction between the ABR and IBR group.

## Highlights

• Bilateral breast reconstruction was more common with alloplastic implantation.
• Complications were more frequent with bilateral than unilateral reconstruction.
• The mean satisfaction score with implanted breast was 43.5 for the autologous group.
• The mean score with implanted breast and 39.6 for the implant-based group.

## 1 Introduction

Breast cancer is one of the most prevalent cancers worldwide. According to the statistics of the Centers of Disease Control and Prevention in 2015, breast cancer is the most frequently diagnosed cancer of all cancer diagnoses, and it is the second most cause of death from cancer after lung cancer [1]. Another study conducted in Saudi Arabia showed that an increase in the prevalence rate of breast cancer, especially among younger patients [2]. According to a research that examined the epidemiology of breast cancer in many Arabic countries, in Saudi Arabia, the most common presentation is advanced breast cancer and the most commonly performed surgery is total mastectomy [3].

With advancements in and evolution of the medical field, there are currently several methods and surgical techniques for breast reconstruction after mastectomy. Generally, we can categorize them into two broad groups: autologous reconstruction and implant-based reconstruction. The implant-based reconstruction could be a single- or multiple-stage reconstruction. Breast reconstruction can be performed immediately after mastectomy or later [4].

Immediate reconstruction, after the breast cancer is properly treated, has been approved, as this enhances the psychiatric well-being of patients after the operation. Additionally, when comparing patients who underwent mastectomy without reconstruction and those who undergo successful reconstruction, patients in the reconstructive group were significantly more satisfied with their quality of life as well as their physical and sexual functionality [5,6].

In this study, we analyzed the health-related quality of life of patients who underwent breast reconstruction surgery by using the BREAST-Q questionnaire, which is a well-designed and validated questionnaire that has been used in several international studies. Patient-reported outcomes (PROs) are one of the subjective measurements that may help in providing useful insights about patients [7]. To our knowledge, this study is the first to be conducted in Saudi Arabia, and hopefully, it will provide a useful perspective about patient feelings and whether patients regret their choices.

## 2 Materials and methods

### 2.1 Study population and design

This is a prospective, cross-sectional study that analyzes breast cancer patients who underwent mastectomy and subsequent breast reconstruction at our institute between August 1, 2013, and August 31, 2017. A questionnaire was distributed manually during the outpatient department (OPD) visits at separate breast reconstructive surgery clinics and was to be completed during the visit or at home and then submitted to us later. Aiming to analyze the quality of their lives, we followed the Arabic version of the BREAST-Q questionnaire reconstruction module (postoperative), which covers two domains: satisfaction with breast reconstruction and satisfaction with the surgical outcomes. We also provided an electronic version of the questionnaire, providing the patients who wished to complete it at home a web link.

BREAST-Q is a self-administered questionnaire, and thus, the recall bias had to be reduced by excluding patients who were illiterate, and if the surgery exceeded 48 months, the patients were not eligible to partake in the study, as enough time is need for healing and recovery from surgery and reporting on one's satisfaction. Moreover, this time should not be too long to prevent recall bias [8]. Questionnaire were given to patients in different interval starting from 6 months to 3 years postoperative. Cases of partial mastectomies and patients with aesthetic breast reconstruction were also excluded. Patient data were obtained from the hospital's medical database, including their age, body mass index, type of reconstructive surgery, laterality, timing of the surgery, preoperative chemotherapy, and radiotherapy.

All breast reconstructive surgeries were done by single plastic surgeon using same surgical technique for each surgical procedure. Major complication where considered when there was secondary surgical intervention such as, partial flap necrosis needed debridement or hematoma that need evacuation.

After the participants had completed the BREAST-Q questionnaire, the raw data were extracted and plotted on an excel sheet to score it on the Q-score system, which revealed a satisfaction score from 0 (not satisfied) to 100 (very satisfied). This score was used in the statistical analysis.

This study was approved by the Institutional Review Board of King Fahad Specialist Hospital in Dammam, Saudi Arabia (IRB study number: SUR0318). This study was reported in line with the STROCSS criteria [9]. And register in open access database (UIN: researchregistry4491).

### 2.2 Breast-Q questionnaire

It's an instrument designed to evaluate outcomes among women undergoing breast surgery. There are five modules (Augmentation, reduction/mastopexy, mastectomy, reconstruction, breast conserving surgery) each comprised of multiple scales. The conceptual framework of BREAST-Q modules based on two themes, patient satisfaction and health-related quality of life. Each scale is designed to function independently. Patients can thus be asked to complete some or all of a module's scales. In this study we used the reconstructive domain specifically, the satisfaction with breast and satisfaction with outcomes scales. Detailed data of the survey question is mention in (Table 1).

**Table 1:** BREAST-Q™ - reconstruction module.

**Satisfaction with breasts (answers range from 1 to 4 points)**
How you look in the mirror clothed?
The shape of your reconstructed breast when you are wearing a bra?
How normal you feel in your clothes?
The size of your reconstructed breast?
Being able to wear clothing that is more fitted?
How your breasts are lined up in relation to each other?
How comfortably your breasts fit?
The softness of your reconstructed breast?
How equal in size your breasts are to each other?
How natural your reconstructed breast looks?
How naturally your reconstructed breast sits/hangs?
How your reconstructed breasts feel to the touch?
How much your reconstructed breast feels like a natural part of your body?
How closely matched your breasts are to each other?
How your reconstructed breast looks now compared to before you had any breast surgery?
How you look in the mirror unclothed?
**Satisfaction with outcome (answers range from 1 to 4 points) implant**
The amount of rippling (wrinkling) of your implant that you can see?
The amount of rippling (wrinkling) of your implant that you can feel?
**satisfaction with outcome (answers range from 1 to 3 points) surgery**
Having reconstruction is much better than the alternative of having no breast?
I would encourage other women in my situation to have breast reconstructive Surgery?
I would do it again?
I have no regrets about having the surgery?
Having this surgery changed my life for the better?
The outcome perfectly matched my expectations?
It turned out exactly as I have planned?

### 2.3 Statistical analyses

Descriptive analysis was used to describe numeric variables. Student's t-test and Mann-Whitney *U* test were used to compare numeric variables in normally distributed data and non-normally distributed data, respectively. To compare between categorical groups, the chi-square test was used. The statistical tests were used as appropriate. The data were analyzed using the SPSS Version 25.

## 3 Results

Forty-three out of 61 patients completed the questionnaire and were included in the study. The response rate for BREAST-Q was 70.5%; those were then divided into two groups: autologous breast reconstruction (ABR) group (n = 21) and implant-based breast reconstruction (IBR) group (n = 22). The internal consistency of the data is significantly high (Cronbach's alpha: 0.751). The ABR group surgery were generally done by either Transverse rectus abdominis myocutaneous (TRAM) pedicled flap or latissimus dorsi (LD) flap. Whereas IBR group it was either immediate reconstruction by silicone implant or staged reconstruction with tissue expander then breast implant.

Many differences were noted and summarized between the two groups (Table 2). With respect to age, the mean age in the two groups were fairly similar; however, patients in the ARB group were slightly older than those in the IBR group, with a mean of 46 and 40, respectively. Moreover, BMI was almost equal between the two groups, 30.8 kg/m^2^ for ABR and 29.7 kg/m^2^ IBR. Interestingly, the bilateral breast reconstruction was significantly performed with alloplastic implantation techniques (*p* < 0.05), and all ABR patients underwent unilateral breast reconstruction. There was no significant difference between both groups in receiving pre-operative chemotherapy or radiotherapy. Major complications were present in eight women on both groups (38% and 36% for ABR and IBR, respectively).

**Table 2:** Clinical data of patients and demographics.

	Autologous	Implant-based	*P*-value
No.	21	22	
Age	46.19 ± 9.1	40.95 ± 10.4	0.101
BMI	30.76 ± 5.4	29.74 ± 6.7	0.395
BMI > 30			0.287
Yes	12 (57)	13 (59)	
No	9 (43)	9 (41)	
Laterality			0.021^a^
Unilateral	21 (100)	16 (72)	
Bilateral	0 (0)	6 (28)	
Timing of surgery			0.287
Immediate	9 (43)	13 (59)	
Delay	12 (57)	9 (41)	
Radiotherapy			0.897
Yes	12 (57)	13 (59)	
No	9 (43)	9 (41)	
Chemotherapy			1.000
Yes	17 (81)	17 (77)	
No	4 (19)	5 (23)	
Major complication			0.907
Yes	8 (38)	8 (36)	
No	13 (62)	14 (64)	

BMI, body mass index.

a A p-value of < 0.05 was considered statistically significant.

However, the BREAST-Q satisfaction questionnaire showed no significant difference between the two groups. The mean satisfaction score for ABR was 43.5 for satisfaction with breast and 45.4 for the satisfaction with surgical outcomes, whereas for the IBR satisfaction with breast was 39.5 and 56.0 for the outcome (Table 3).

**Table 3:** Mean scores of the BREAST-Q questionnaire between ABR and IBR and the statistical comparison between them.

	Autologous	Implant-based	*p*-value
Satisfaction with breast	43.52 (29.16)	39.59 (17.34)	0.597
Satisfaction with surgical outcome	45.43 (36.58)	56.00 (31.33)	0.378

We found that complications occurred more frequently with bilateral reconstruction (83%) than with unilateral reconstruction (30%; *p* = 0.021). All patients who underwent bilateral reconstruction were from the IBR group (n = 6).

## 4 Discussion

In the past, PROs have enabled a reliable analysis of the postoperative quality of life and general satisfaction of patients. PROs provide an insight into surgical outcomes from the patient's perspective rather than from the surgeon's. PRO is one of the important assessment methods in the surgical field to improve patient-centered care [ 10,11]. The BREAST-Q questionnaire is one of the most reliable, validated, and effective tools to study the satisfaction rate in multiple domains: satisfaction with breast, surgical outcome, physical well-being, and the surgeon. Additionally, this survey has multiple versions of procedure-specific module, such as reconstruction, mastectomy, augmentation etc., which are available in several languages [12].

Multiple studies conducted in different countries have shown a significant difference between mastectomy alone without reconstruction and breast reconstruction either with implant or autologous reconstruction in regard to the quality of life and satisfaction. The results encourage breast cancer patients to have their breast reconstructed for a better quality of life and greater satisfaction [[13], [14], [15], [16]]. Howes et al. showed that breast-conserving surgeries have been found to be associated with lower physical well-being and quality of life following breast reconstruction. They also conclude that mastectomy without reconstruction has the lowest score of satisfaction between their three study groups [17]. With all these studies confirming that breast reconstruction is a major component in completing the treatment circle of breast cancer patients, we should standardize the reconstruction surgery for all patients and determine which reconstruction is suitable for individual patients, considering the patient's perspective.

Our results are similar to those of other studies, highlighting no statistically significant difference in the quality of life or general satisfaction between the two groups [[18], [19], [20], [21]]. It has been highlighted that not only does the type of reconstruction affect the quality of life, but also the mentality, expectation, and pre-operative psychological status of patients can affect their decisions and subsequent satisfaction [22]. Body image perception and quality of life were found significantly lower in patients diagnosed with malignant breast cancer than in those with benign breast lesions after successful breast reconstruction [23].

In summary, this study was designed to determine the best surgical procedure for breast reconstruction after mastectomy. Many studies have examined the patient's perspective in a single category for breast reconstruction. For example, Dieterich et al. found that patients in whom breast reconstruction involved implants plus mesh have a higher satisfaction with the surgeon and staff rather than those with implant alone [24]. McCarthy et al. conducted a study comparing silicone and saline implants for patients who underwent mastectomy and subsequent reconstruction, and they found that silicone implants had a higher satisfaction outcome than did saline implants [25]. Drazan et al. reached the conclusion that around 92% of patients with bilateral breast reconstruction have a satisfaction of excellent or good, and they suggest that deep inferior epigastric perforator flap is a better option for bilateral breast reconstruction [26]. Brennan et al. showed that most women who underwent immediate or two-stage breast reconstruction with implant or expander and planned to have postoperative radiotherapy, preserved their implants, with only about 30% needing to convert to autologous reconstruction.

For breast reconstruction, there is a wide spectrum of surgical techniques and methods that range from simple to very sophisticated procedures. Each patient is unique, and the most suitable surgical reconstruction methods should be selected carefully, considering the patient's perspective. There is no such thing called the “ideal” method for reconstruction that is suitable for all breast cancer patients after mastectomy. Pinel-Giroux et al. have described almost all possible methods for breast reconstruction with detailed data that help to understand each method better [27].

The drawback of this study is the small sample size and that the population of the study was from a single institute. We also did not consider the breast cancer type and stage and other patient characteristics, such as smoking, marital status, and date of diagnosis, which may have psychosocial impact on patient satisfaction.

Despite these limitations, this study remains unique since it is the only study in Saudi Arabia analyzing the satisfaction of patients after surgery, highlighting multiple factors that may play a role in affecting the patient's quality of life, such as postoperative chemotherapy and radiotherapy.

The future plan is to increase the sample size and include multiple institutes for more significant and insightful results. Furthermore, we should include the BREAST-Q modules of satisfaction with physical and sexual well-being, as well as satisfaction with the surgeon, medical staff, and the nipples. Examining the psychosocial status before and after breast reconstruction may reveal a more powerful and reliable data.

## 5 Conclusions

Although there are a variety of options for breast reconstruction, there is no single optimal method suitable for all patients. Notably, patient satisfaction tends to be individual-specific because several factors can contribute toward reducing patient satisfaction. In our study, the mean satisfaction score for ABR was higher for satisfaction with breast and lower for the satisfaction with surgical outcomes, in compare with the IBR group. In conclusion, we found that there was no statistically significant difference in satisfaction between the ABR and IBR group as reported in many similar studies in the existing literature.

## Ethical approval

Ethical approval was obtained from the Institutional Review Board (IRB) of the King Fahad Specialist Hospital, Dammam, Saudi Arabia. The ethical approval was issued an IRB Study Number – SUR0318.

## Author contribution

SMA, MYD, KM, AM, GK, MY, and HA participated in study conceptualization, study design, data collection, and literature search. Data were analyzed by SMA and GK. SMA and MYD drafted the manuscript. All authors read and approved the final manuscript.

## Conflicts of interest

The authors declare no conflict of interest.

## Trial registry number

UIN: 4491.

## Guarantor

Salem Mohammad Alshammari.

## Availability of data and materials

The data that support the findings of this study are available from the corresponding author upon reasonable request.

## Provenance and peer review

Not commissioned, externally peer reviewed.

## Sources of funding

This study did not receive any funding from governmental or private organizations.
